# Factors Affecting the Synthesis of Cellobiose Lipids by *Sporisorium scitamineum*

**DOI:** 10.3389/fbioe.2020.555647

**Published:** 2020-11-04

**Authors:** Amira Oraby, Nicole Werner, Zehra Sungur, Susanne Zibek

**Affiliations:** ^1^Fraunhofer Institute for Interfacial Engineering and Biotechnology IGB, Stuttgart, Germany; ^2^Institute of Interfacial Process Engineering and Plasma Technology IGVP, University of Stuttgart, Stuttgart, Germany

**Keywords:** cellobiose lipids, *Sporisorium scitamineum*, fermentation process, glycolipids, biosurfactant

## Abstract

Cellobiose lipids (CL) are extracellular glycolipids that are produced by many microorganisms from the family *Ustilaginaceae*. The sugarcane smut fungus *Sporisorium scitamineum* has been long known as a producer of the glycolipids mannosylerythritol lipids (MEL) and was recently described to additionally secrete CL as a byproduct. In fact, we identified 11 homologous genes in *S. scitamineum* by *in silico* analysis sharing a high similarity to the CL biosynthesis gene cluster of *Ustilago maydis*. We here report the first systematic cultivation of *S. scitamineum* targeting the synthesis of CL with high product titers and its transfer to the bioreactor. In an initial screening we examined different fermentation media compositions, consisting of a mineral salts solution with vitamins and/or trace elements, three carbon sources (glucose, fructose, sucrose), three pH values (2.5, 4.0, 6.7) and three levels of C/N values (42.2, 83.8, 167.2 mol_C_⋅mol_N_^–1^) with urea as nitrogen source. A pH of 2.5 proved to result in the highest product titers. An increase of urea concentration from 0.6 to 1.2 g⋅L^–1^ had a positive effect on biomass formation, however the glycolipid formation was favored at a C/N ratio of 83.8 mol_C_⋅mol_N_^–1^, using 0.6 g⋅L^–1^ urea. Amongst the examined carbon sources, sucrose resulted in an increase in the secretion of cellobiose lipids, compared to glucose. Comparing different media compositions, vitamins were identified as not necessary for CL synthesis. We obtained a concentration of cellobiose lipids of 8.3 ± 1.0 g⋅L^–1^ in shaking flasks. This increased to 17.6 g⋅L^–1^ in the 1 L bioreactor with additional feeding of carbon source, with a final purity of 85–93%. As a side product, erythritol and mannosylerythritol lipids (MEL) were also synthesized. Via HPTLC coupled MALDI-TOF MS we were able to analyze the secreted CL structures. *S. scitamineum* produces a mixture of acylated low molecular weight D-glucolipids, linked to a 2,15,16-trihydroxy-hexadecanoic acid via their ω-hydroxyl group (CL-B). The produced cellobiose lipids precipitate as needle like crystals at an acidic pH value of 2.5.

## Introduction

Cellobiose lipids (CL) are a group of microbial biosurfactants that are secreted as secondary metabolites by many microorganisms from the family *Ustilaginaceae*, with *Ustilago maydis* being the most examined producer of CL. They were first discovered in 1950 by Haskins while screening a wide range of fungi for their ability to metabolize glucose and agricultural wastes (Haskins, 1950). CLs are reported to have various antimicrobial and antifungal activities, as well as gelling characteristics, making them of high interest for application in cosmetics, as detergents, or fungicides (Haskins and Thorn, 1951; Puchkov et al., 2002; Cheng et al., 2003; Teichmann et al., 2007; Imura et al., 2014). Widely studied producers of CL besides *Ustilago maydis* are *Anthracocystis flocculosa* (formerly known as *Pseudozyma flocculosa*), *Kalmanozyma fusiformata, Sporisorium graminicola*, and *Cryptococcus humicola*, amongst others (Kulakovskaya et al., 2006; Golubev et al., 2008; Kulakovskaya et al., 2007; Teichmann et al., 2011a).

**FIGURE 1:**
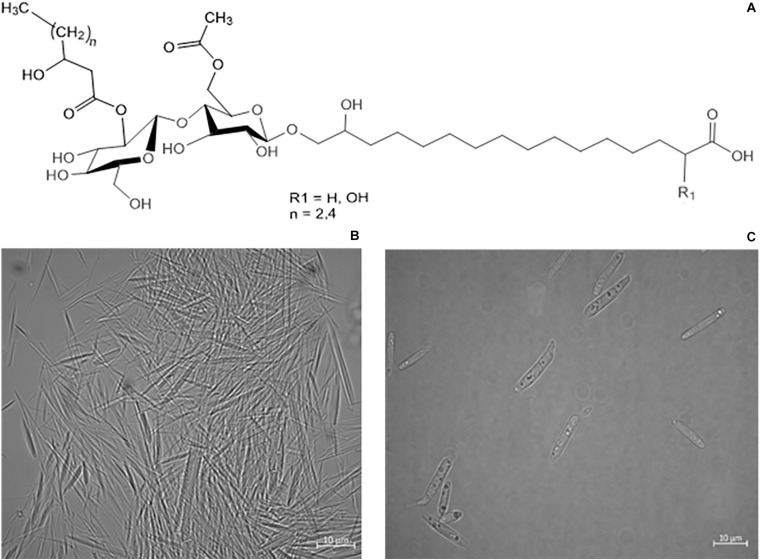
**(A)** Structure variants of CL-B (Spoeckner et al., 1999). **(B)** Microscope images of precipitated needle like CL crystals, secreted by *S. scitamineum.* **(C)** *S. scitamineum* grown in complex YM medium without CL crystals.

CLs are usually produced as a mixture of different acylated low molecular weight D-glucolipids, linked to a hydroxypalmitic acid via their ω-hydroxyl group (Eveleigh and Dateo, 1964). Depending on the producing microorganisms, typical strain-associated structural varieties can be observed. *U. maydis* secretes a CL variant with binding a 15,16-dihydroxyhexadecanoic acid or a 2,15,16-trihydroxy-hexadecanoic acid fatty acid chain to the cellobiose CL-B. The fatty acids can further differ in the presence or absence of their hydroxyl group (R_1_ = H or OH) or the length of the acyl chain at 2″position (Figure 1A; Spoeckner et al., 1999; Teichmann et al., 2007). An additional variant with an ester group is known as CL-C (Spoeckner et al., 1999). *A. flocculosa* produces flocculosin, a CL that has an extra acetyl-group at C3″ position and whose cellobiose is esterified with 2-hydroxy-octanoic acid and acetylated at two positions (Teichmann et al., 2011a). *C. humicola* secretes a mixture of different types of CL with the bolaform 16-*O*-(2″,3″,4″,6′-tetra-*O*-acetyl-ß-cellobiosyl)-2-hydroxyhexadecanoic acid being the major product (Puchkov et al., 2002; Morita et al., 2011). In CL produced by *K. fusiformata* a 2,15,16-trihydroxypalmitic acid is linked to the cellobiose and 3-hydroxycaproic acid and acetic acid are linked as O-acylic substituents, corresponding to the structure of CL-B produced by *U. maydis* (Kulakovskaya et al., 2005).

A gene cluster containing 12 open reading frames coding for enzymes needed for CL synthesis was first identified in *U. maydis*, proposing a biosynthesis route for CL (Teichmann et al., 2007). In *U. maydis*, the zinc finger transcription factor Rua1 regulates CL synthesis (Teichmann et al., 2010). The monooxygenase Cyp1 terminally hydroxylates the *de novo* synthesized precursor palmitic acid, while Cyp2 ω-1 hydroxylates the 16-OH palmitic acid. The two glucose units of the sugar moiety are added by the glycosyltransferase Ugt1. Fas2 synthesizes the additional fatty acids and Uhd1 catalyzes their β-hydroxylation. The acetyltransferase Uat2 transfers the shorter fatty acid to CL in C6′ position, while Uat1 acylates the C2″ position in the cellobiose molecule. Ahd1, the α-hydroxylase, catalyzes the hydroxylation of the palmitic acid. Finally CL is exported by the transporter Atr (Teichmann et al., 2007, 2011a). A highly similar gene cluster with 11 open reading frames was identified for *A. flocculosa*, thus suggesting a highly conserved biosynthesis pathway in both glycolipid producers (Teichmann et al., 2011a).

*Sporisorium scitamineum* (formerly *Ustilago scitaminea*) is another *Ustilaginaceae* species, that is known as sugar cane smut fungus (Braithwaite et al., 2004). Phylogenetic analysis revealed *Sporisorium reilianum* as sister species, both branching early in the evolutionary history from *U. maydis* (Que et al., 2014). However, genomic characteristics like genome and gene size, GC content or number of exons and introns etc. are most similar to *U. maydis* (Que et al., 2014; Taniguti et al., 2015; Dutheil et al., 2016). Besides, encoded proteins in *S. scitamineum* show an average of 75.4% identity to proteins of *U. maydis* (Que et al., 2014), which confirms their relationship. *S. scitamineum* (NBRC 32730) was long known as MEL producer, another biosurfactant that is also produced by. *U. maydis* strains (Hewald et al., 2006; Morita et al., 2009a,b). In 2014 the first synthesis of CL by *S. scitamineum* (NCBI: txid49012) was reported, as a result of a wide screening of different *Ustilaginaceae* species for the production of value-added chemicals (Geiser et al., 2014). The produced structures are correspondent to CL variants produced by *U. maydis* (Deinzer, 2017). However, to our knowledge no further studies on CL synthesis by *S. scitamineum* are published, and the sequences of the CL biosynthesis cluster have not yet been described. While CL is of potential interest for application in various industrial sectors, as previously mentioned, fundamental research is still needed for a better understanding of factors affecting CL synthesis. A research gap is evident here.

Therefore, this paper aims to study *S. scitamineum* as a CL producer and analyse factors potentially important for CL synthesis. As *S. scitamineum* is closely related to *U. maydis*, we hypothesized that factors affecting its glycolipid production are similar. In order to gain a better understanding and to verify this hypothesis, the effect of pH value, carbon source and C/N ratio as well as media composition on microbial growth and CL synthesis were examined. All these factors were reported to have direct effects on the produced amount of CL amongst the various producing microorganisms (Roxburgh and Spencer, 1954; Hammami et al., 2008; Günther et al., 2010; Liu et al., 2011; Zavala-Moreno et al., 2014). Furthermore, most studies related to microbial CL synthesis focus on shaking flask cultivations, with only limited publications reporting a CL production in bioreactors, with titers varying from 13 to 33 g⋅L^–1^ (Roxburgh and Spencer, 1954; Frautz et al., 1986; Liu et al., 2011; Morita et al., 2011). To generate more process knowledge for CL fermentations in bioreactors, we further present the first cultivation of *S. scitamineum* for CL synthesis in a fermenter.

## Materials and Methods

### Protein Sequence Homology Analysis of *S. scitamineum*

Protein sequences of the *U. maydis* (NCBI: txid237631) CL gene cluster were used to identify open reading frames and find homologous sequences in the *S. scitamineum* (NCBI: txid1447027) annotated genome (ASM90000236v1) (Dutheil et al., 2016) by alignment analysis via BLAST. This strain is from the same clade as used for our experiments. Amino acid sequence similarities and identities were calculated using the Needle EMBOSS software (Madeira et al., 2019).

### Strain and Seed Culture

Glycerol cryo cultures of the strain *Sporisorium scitamineum* (*Ustilago scitaminea*) DSM 11941, obtained from the German Collection of Microorganisms and Cell Cultures (DSMZ), were stored at −80°C and used to streak agar plates containing YM medium (10 g⋅L^–1^ glucose, 5 g⋅L^–1^ peptone, 3 g⋅L^–1^ malt extract, 3 g⋅L^–1^ yeast extract and 15 g⋅L^–1^ agar (DSMZ, 2020)). The pH was adjusted to pH 6 using 2 M H_2_SO_4_. These plates were incubated for 48 h at 30°C and kept for up to 30 days at 8°C.

For seed culture fermentation, strains were obtained from agar plates and cultivated in liquid YM medium, pH 6, at 30°C and 120 rpm in a rotary shaker. After a maximum of ∼17 h, 1 L baffled shaking flasks containing 200 mL of YM medium were inoculated to an optical density (OD_625_) of 0.1 a.u. and cultivated under the same conditions until the glucose concentration in the medium decreased to less than 1 g⋅L^–1^. This seed culture was used to inoculate CL production culture in further experiments.

### Fermentation Strategy for CL Production

For CL fermentation, basic production culture medium (PCM), based on the composition of YNB (Y1251 Merck; Germany), consisting of mineral salts (1.0 g⋅L^–1^ KH_2_PO_4_, 0.5 g⋅L^–1^ MgSO_4_, 0.1 g⋅L^–1^ NaCl, 0.1 g⋅L^–1^ CaCl_2_), compounds supplying trace elements (500 μg⋅L^–1^ H_3_BO_3_, 40 μg⋅L^–1^ CuSO_4_, 100 μg⋅L^–1^ KI, 200 μg⋅L^–1^ FeCl_3_, 400 μg⋅L^–1^ MnSO_4_, 200 μg⋅L^–1^ Na_2_MoO_4_, 400 μg⋅L^–1^ ZnSO_4_) and a vitamin solution (2 μg⋅L^–1^ biotin, 400 μg⋅L^–1^ calcium pantothenate, 2 μg⋅L^–1^ folic acid, 2 mg⋅L^–1^ inositol, 400 μg⋅L^–1^ niacin, 200 μg⋅L^–1^ p-aminobenzoic acid, 400 μg⋅L^–1^ pyridoxine hydrochloride, 200 μg⋅L^–1^ riboflavin, 400 μg⋅L^–1^ thiamin hydrochloride), was used (Günther et al., 2010). Unless otherwise indicated, 0.6 g⋅L^–1^ urea and 50 g⋅L^–1^ glucose were added to the medium and inoculated to an OD_625_ of 0.3 a.u. from the previously prepared seed culture. All fermentations were conducted at 30°C and an initial pH of 2.5 for 10–14 days, unless differently indicated (method optimized from Günther et al., 2010).

Fermentations in micro-bioreactor systems were performed in the BioLector I (m2p Labs GmbH; Germany) in 48-well Flowerplates^®^ (m2p-labs) with online monitoring of dissolved oxygen (DO) and backscatter (measured by backscattered light at λ = 620 nm, gain 10) (Samorski et al., 2005; Funke et al., 2009). Data points were measured every 20 min. For growth rate calculations, the moving averages of 50 data points of the backscatter signal were used to reduce noise. The wells were filled up to 1,300 μL and shaken at 800 rpm. Cultivations were performed in duplicates or triplicates. Cell dry weight (CDW), CL concentration and concentration of remaining sugars were measured offline after termination of the fermentation.

Fermentations in shaking flasks were performed in 1 L baffled flasks with 200 mL medium at 120 rpm in a rotary shaker. 1 mL samples were taken every 24 h for monitoring of OD, CDW, CL concentration as well as nitrogen and carbon source concentrations.

For bioreactor fermentations 700 mL of fermentation media were inoculated in 1 L INFORS HT Multifors bioreactors (Infors AG; Germany) with online monitoring of DO, pH, stirrer speed and T. The reactors were aerated with 1 vvm (volume of gas per volume of liquid per minute) of air and stirred within a range of 400–500 rpm, to maintain a DO value above 20%. Two fermenters were started in parallel to ensure reproducibility of the fermentation. 1 mL samples were taken every 24 h for offline monitoring of OD, CDW, CL concentration as well as nitrogen and carbon source concentrations.

### Variation of Fermentation Parameters and Media Compositions

The effect on growth and glycolipid formation of three different pH values (2.5, 4.0, and 6.7) was examined. This pH range was chosen for our screening, since acidic pH values were described to result in an increase in CL production rate with *U. maydis* (Günther et al., 2010). Furthermore the transfer phase of seed culture to the production culture medium was done during the early or late stationary phase, to assess a potential effect of substrate limitation of *S. scitamineum* on their CL productivity. Three different carbon sources were used for fermentation, at a concentration of 50 g⋅L^–1^: sucrose, glucose and fructose. All three carbon sources can be metabolized by *S. scitamineum* (NBRC 32730) for glycolipid production (Morita et al., 2009a, 2015). 50 g⋅L^–1^ glucose + 0.6 g⋅L^–1^ urea [(IIa) 83.8 mol_C_⋅mol_N_^–1^] was chosen based on Spoeckner and Günther as starting/basic C/N ratio, showing the best results regarding CL concentrations with *U. maydis* (Spoeckner et al., 1999; Günther et al., 2010). We further examined the possible effect of halved [(I) 42.2 mol_C_⋅mol_N_^–1^] and doubled [(III) 167.2 mol_C_⋅mol_N_^–1^] C/N ratios on CL formation and biomass growth. The C/N ratio (II) was further applied at two concentration levels (IIa) and (IIb). The equivalent glucose and urea concentrations are summarized in Table 2 in the “Results” section.

To assess the effect of different media constituents on CL productivity and biomass growth, *S. scitamineum* was cultivated on the different fractions of PCM [mineral salts, vitamins (Vit), compounds supplying trace elements (TE)] separately, with or without an addition of 0.01 g⋅L^–1^ FeSO_4_, corresponding to an increase by ∼54 mol in iron ions. All used media combinations are summarized in Table 2.

### Analytical Methods

For biomass quantification, the optical density was measured photometrically at 625 nm (GENESYS^TM^ 10 UV, Thermo Fisher Scientific; United States). For all further analyses, 1 mL sample was centrifuged at 16,060 g for 10 min at RT. The supernatant was used for urea and sugar analysis. Urea concentration was determined photometrically, via enzymatic reaction with a urea/ammonia test kit (R-Biopharm; Germany). Sugar concentration was measured via HPLC with a Supelcogel 8 H 59246-U column (Merck; Germany), at 30°C with 5 mM H_2_SO_4_ as mobile phase, with a flow rate of 0.6 mL⋅min^–1^ and a running time of 15 min. Sucrose, glucose, fructose and erythritol were detected via a refractive index detector (RI 8120, Bischoff GmbH; Germany).

The pellet was washed with acidic water (pH 2; H_2_SO_4_) to remove residual sugars, then 1 mL of ethanol was added to the pellet for CL extraction. The extraction with 1 mL ethanol was repeated and both extracts were unified and stored at −18°C until further analysis (Günther, 2014). The remaining pellet after ethanol extraction was dried and used for gravimetric CDW determination.

For CL quantification, the ethanol extracts were applied to HPTLC Silica gel 60 F_254_ plates (Merck; Germany) in a chloroform-methanol-water (65:25:4) system. The spots were visualized by dipping into a developing solution (mixture of acetic acid, sulfuric acid and anisaldehyde) and heating until color intensity stabilized. The spots intensities were then quantified with the software Image-J and the CL concentrations calculated based on the internal calibration with CL standards.

For obtaining the standards, CL was extracted with ethanol, as previously described, from the pellet of 25 mL *S. scitamineum* culture broth. The extract was evaporated at 45°C and the obtained white/yellowish CL was grinded to a powder. This CL fraction was suspended twice in 4 mL of ethyl acetate per g CL and incubated for 30 min at RT to remove remaining fatty acids and MEL (Günther, 2014). The suspension was then centrifuged for 10 min at 16,060 g and 4°C and the pellet containing the purified CL was dried at 45°C and grinded to a powder. The purity of the standard was determined densitometrically via HPTLC as 91.6 ± 2.9% (*n* = 4). This purified CL was dissolved in ethanol, at concentrations of 10, 5, 2.5, and 1.25 g⋅L^–1^ and used as standard for internal calibration. Values from different plates are referred to the mean of sample PCM (IIa) for proper comparison.

For structural analysis a matrix consisting of 200 g⋅L^–1^ dihydroxybenzoic acid, 1.15 g⋅L^–1^ ammonium hydrogen phosphate, 0.1 g/L Octyl-β-D-glycopyranoside, 0.1% (v/v) trifluoroacetic acid in 90% acetonitrile and 10% ddH_2_O was applied to the HPTLC plates. Polypropylene glycol was spotted on the plate as internal standard to verify the mass calibration of the MS system. The plates were then dried overnight in a desiccator, prior to sample application for the MALDI-TOF-MS measurement. Then they were developed analogous to the method for CL quantification and separated due to polarity, however without treatment with the developing solution. Bands were scanned over their complete running distance with Bruker Ultraflex II TOF/TOF controlled by flex Control software (Bruker Daltonics; United States). Spectra were calculated in the range of 200–2,000 Da (m⋅z^–1^) based on the mean value of five spots at a distance of 0.6 mm in the running direction, with 200 laser shots for each. The obtained data was analyzed with flex Analysis and TLC MALDI software (Bruker Daltonics; United States), and the detected masses of the [M + Na]^+^ adducts were compared with known CL and MEL molecular masses from *U. maydis* (Spoeckner et al., 1999; Teichmann et al., 2011a; Beck et al., 2019). In combination with the polarity pattern, probable CL structures were derived from the mass spectrum of each spot. Only masses with an intensity above 500 a.u. (threshold) were considered. The m/z resolution was 1 Da. Therefore, a constant pattern of the isotopic distribution could be obtained for each spot and was considered for data analysis. Each detected mass spot consists of three peaks, with a mass difference of 1 Da and diminishing relative abundance, explained by the natural isotopic distribution. This TLC coupled MALDI-TOF-MS method was first described for CL analysis by Günther and recently published in detail by Günther (2014) and Beck et al. (2019).

## Results and Discussion

Examinations with different media compositions and pH values were conducted in the micro-bioreactor system. Observations on CL formation kinetics were either based on shaking flask or bioreactor experiments.

### Comparative Genome Analysis of the CL Gene Cluster in *U. maydis* and *S. scitamineum*

The CL gene cluster is highly preserved amongst the known and sequenced CL producers *U. maydis and A. flocculosa.* Although *A. flocculosa* shares only an average gene sequence identity of about 50% with *U. maydis*, their CL-clusters share high sequence identities (up to 97%). Only the homolog for Rua1 shares a low identity of 23% (Teichmann et al., 2007, 2011a; Morita et al., 2013; Dutheil et al., 2016). Cyp 1, a gene encoding a P450 oxidoreductase, which is essential for CL synthesis, is also confirmed to be present in other strains that produce CL, such as *M. aphidis* and *P. hubeiensis pro tem* (Morita et al., 2013).

**FIGURE 2:**
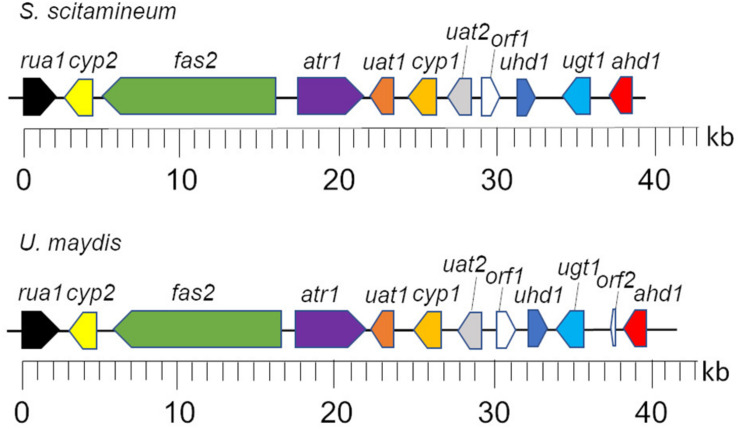
Genetic organization of the CL biosynthesis cluster of *S. scitamineum* and *U. maydis*. Gene designations are described in Table 1. Figure was modified after Teichmann et al. (2011a).

In the clade *S. scitamineum*, which is known to produce both MEL and CL, a homolog to the sequenced MEL biosynthesis gene cluster was identified recently (Deinzer, 2017). Furthermore, CL structures obtained from *U. maydis* and *S. scitamineum* share a high structural identity. Therefore it was of a high probability to find a homologous CL gene cluster in *S. scitamineum* as well. Via sequence homology analysis, we scanned the public available genome of *S. scitamineum* (Dutheil et al., 2016) and were able to identify 11 out of 12 clustered homologs to the genes associated with CL biosynthesis in *U. maydis* (Table 1). All 11 genes are located on scaffold 33 (NCBI accession number LK056681) with a spanning region of ∼40 kb in identical genetic organization as in *U. maydis* (Figure 2) and with high similarities varying between 63 and 89% to the *U. maydis* homologs, except for Rua1, that shares only 51% similarity.

**TABLE 1:** Homologous genes of CL biosynthesis gene cluster in *S. scitamineum*. Amino acid (AA) sequence similarity was calculated via the EMBOSS Needle software (Madeira et al., 2019).

*U. maydis* 521	Description	Accession No	Gene sequence length (bp)	Homolog in *S. scitamineum* (Accesion-No)	Description	Gene sequence length (bp)	Similarity (AA)	Identity (AA)
cyp1	Cytochrome P450 enzyme invovled in glycolipid production	XP_011392749	1923 (rev)	CDU25005.1	Related to cytochrome P450	1794 (rev)	83%	77%
cyp2	Cytochrome P450 monooxygenase involved in ustilagic acid production	XP_011392727	1608 (rev)	CDS01512.1	Hypothetical protein	1602 (rev)	89%	83%
ugt1	Ustilagic acid glycosyl transferase	XP_011392734	1737 (rev)	CDU25009.1	um12340	1734 (rev)	80%	69%
uat1	Ustilagic acid acyltransferase	XP_011392730	1542 (rev)	CDU25004.1	Probable ustilagic acid acyltransferase	1536 (rev)	80%	69%
fas2	Fatty acid synthase FAS2	XP_011392728	11115 (rev)	CDU25002.1	Probable fatty acid synthase, beta and alpha chains	11121 (rev)	85%	74%
uhd1	Ustilagic acid hydroxylase	XP_011392733	903 (for)	CDU25008.1	Probable ustilagic acid hydroxylase	915 (for)	77%	63%
ahd1	Alpha-hydroxylase AHD1	XP_011392763.1	1151 (for)	CDU25010.1	Probale aplha-hydroxylase AHD1	1140 (for)	88%	77%
rua1	Ustilagic acid biosynthesis regulator rua1	XP_011392726	2274 (for)	CDU25000.1	Related to regulator of ustilagic acid biosynthesis	2052 (for)	51%	39%
atr1	ABC transporter	XP_011392729	4149 (for)	CDU25003.1	Probable ABC transporter	4203 (for)	82%	73%
orf1	Unknown	XP_011392732	1149 (for)	CDU25007.1	Uncharacterized protein	1164 (rev)	80%	64%
uat2	Ustilagic acid acyltransferase	XP_011392731	1290 (for)	CDU25006.1	Uncharacterized protein	1302 (rev)	63%	49%
orf2	Unknown	XP_011392750	348 (rev)	No significant similarity found				

**Rev, reverse orientation; for, forward orientation.**

**TABLE 2:** Used pH values and media compositions for the screening experiment with *S. scitamineum* and obtained CL and CDW concentrations, CL yield and glucose and erythritol concentrations in the medium at the end of fermentation.

Medium composition	c_C__–__s__our__ce_[g⋅L^–^^1^]	c_Urea_[g⋅L^–^^1^]	Inoculum pH	c_CL_ (t = 240 h) [g⋅L^–^^1^]	Y_P/S_(t = 240 h) [g⋅g^–^^1^]	c_CDW_ (t = 240 h) [g⋅L^–^^1^]	c_Glucose_ (t = 240 h) [g⋅L^–^^1^]	c_Erythri__to__l_ (t = 240 h) [g⋅L^–^^1^]
PCM	Glucose: 50	0.6	6.7	0.0 ± 0.0	0.00	4.3 ± 0.2	1.8 ± 0.0	14.3 ± 0.5
PCM	Glucose: 50	0.6	4	0.0 ± 0.0	0.00	5.1 ± 0.4	1.4 ± 0.0	4.2 ± 0.4
PCM (IIa)	Glucose: 50	0.6	2.5	5.1 ± 0.3	0.10	4.6 ± 0.3	0.4 ± 0.0	4.7 ± 0.3
PCM (III)	Glucose: 100	0.6	2.5	4.8 ± 1.2	0.05	5.6 ± 0.2	24.2 ± 0.5	20.2 ± 0.4
PCM (IIb)	Glucose: 100	1.2	2.5	2.1 ± 0.2	0.02	7.5 ± 0.3	1.9 ± 0.0	21.6 ± 0.9
PCM (I)	Glucose: 50	1.2	2.5	<0.5	<0.01	5.4 ± 0.0	1.1 ± 0.1	0.9 ± 0.3
PCM	Fructose: 50	0.6	2.5	6.1 ± 0.1	0.12	4.4 ± 0.1	0.0 ± 0.0	0.3 ± 0.0
PCM	Sucrose: 50	0.6	2.5	6.6 ± 0.2	0.13	4.3 ± 0.3	0.4 ± 0.0	3.1 ± 0.3
PCM + FeSO_4_	Glucose: 50	0.6	2.5	1.4 ± 0.1	0.03	4.6 ± 0.2	1.4 ± 0.1	6.7 ± 0.5
PCM without trace element solution (PCM-TE)	Glucose: 50	0.6	2.5	0.0 ± 0.0	0.00	2.3 ± 0.5	25.0 ± 0.5	4.2 ± 0.4
PCM without trace element solution + FeSO_4_ (PCM-TE + FeSO_4_)	Glucose: 50	0.6	2.5	0.0 ± 0.0	0.00	3.5 ± 0.2	20.0 ± 0.3	3.2 ± 0.2
PCM without vitamin solution (PCM-Vit)	Glucose: 50	0.6	2.5	3.8 ± 0.6	0.08	4.4 ± 0.3	0.4 ± 0.0	4.8 ± 0.2
PCM without vitamin solution + FeSO_4_ (PCM-Vit + FeSO_4_)	Glucose: 50	0.6	2.5	1.5 ± 0.1	0.03	4.5 ± 0.4	1.6 ± 0.3	6.4 ± 0.5
								

However, the potential homolog to Rua1 that we identified in *S. scitamineum*, annotated as CDU25000.1, contains a Cys2His2-motif at the N-terminus that has 92.9% similarity (Supplementary Figure 1). This corresponds to the homology results observed on *U. maydis* and *A. flocculosa*, where only the Cys2His2-motif within Rua1 had a high identity in both microorganisms (Teichmann et al., 2011a).

These results confirmed the expected conservation of the CL biosynthesis pathway in *S. scitamineum* and pave the way for further molecular biological experiments to study gene function and perform strain optimization.

### Observations on the Growth Behavior of *S. scitamineum* in Complex Medium

**FIGURE 3:**
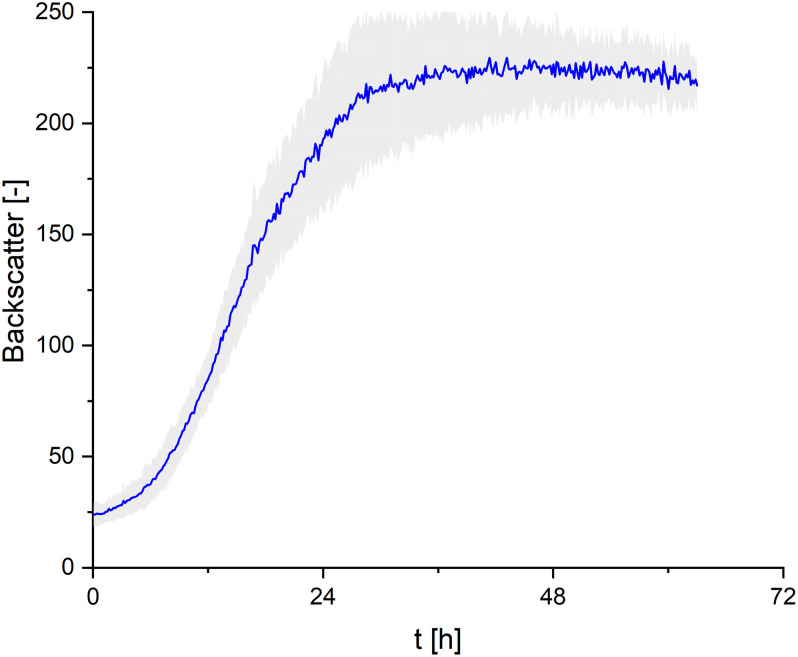
Growth kinetics of *S. scitamineum* seed culture in complex YM medium, cultivated in the micro-bioreactor system at an inoculation pH of 6.0. Error bars are deviated from 8 parallel cultivations (*n* = 8).

In order to cover all nutrients necessary for biomass growth and determine growth kinetics of *S. scitamineum*, seed cultures were grown on the complex YM medium. A maximum growth rate of μ_max_ = 0.15 ± 0.02 h^–1^ was observed within the first 8 h of cultivation at 30°C, pH 6 and 800 rpm in the micro-bioreactor system, while maximum backscatter was reached after ∼ 30 h (Figure 3). However, no CL formation was observed under these conditions in the seed culture (Figure 1C).

When *U. maydis* (DSM 17146) was cultivated with resting cells, i.e., without further growth in the production culture, the biomass transfer point from seed culture to the CL fermentation culture showed an effect on the subsequent CL productivity (Günther et al., 2010). In order to exclude a similar sensitivity with *S. scitamineum*, cell biomass was transferred either at the early (∼28 h) or late stationary phase (∼42 h) from the seed culture to the CL production culture. No significant effect of the biomass transfer phase was observed, neither on the obtained CDW nor on subsequent CL formation, indicating a tolerance of *S. scitamineum* toward substrate limiting conditions (results not shown here). All cultivations for CL production were therefore inoculated from this seed culture during the early stationary phase, at ∼28 h.

### Effect of pH Value

**FIGURE 4:**
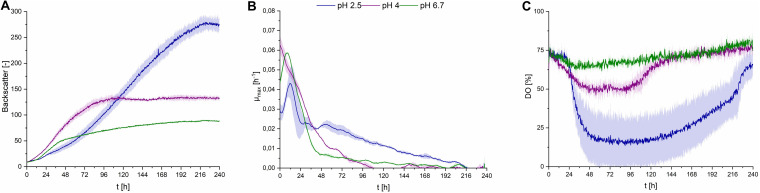
Observations on *S. scitamineum* during fermentation in the micro-bioreactor system, using PCM, 50 g⋅L^–1^ glucose and 0.6 g⋅L^–1^ urea at an inoculation pH of 2.5, 4, or 6.7. **(A)** Backscatter light signal, **(B)** specific growth rates, and **(C)** DO level. Error bars are deviated from biological triplicates (*n* = 3).

Fungal secondary metabolism in general is regulated by various environmental stimuli, including pH (Brakhage, 2013). In order to examine a pH dependency of CL synthesis with *S. scitamineum*, three different pH ranges were used for fermentation in PCM: pH 6.7, the pH level of the used defined PCM without any adjustment, an acidic pH of 2.5 and pH 4.0. At pH 6.7 the worst growth behavior amongst the examined pH ranges was observed (Figure 4A). A biomass concentration as low as 4.3 ± 0.2 g⋅L^–1^ was obtained, while no CL was detected (Table 2). At pH 4 backscatter and growth rate (Figures 4A,B) were slightly higher over the course of fermentation, with a c_CDW_ of 5.1 ± 0.4 g⋅L^–1^. However, no CL was produced here either, compared to pH 2.5 where we obtained a c_CDW_ of 4.6 ± 0.3 g⋅L^–1^ and a c_CL_ of 5.1 ± 0.3 g⋅L^–1^.

While at pH 2.5 the lag phase was longest and the maximum growth rate μ_max_ was lowest, an increase in optical density was observed up to 216 h of fermentation, showing a stable growth over a longer time span. This was also reflected in the DO level (Figure 4C), where it decreased to lower than 25% after an initial lag phase. At pH 4 and pH 6.7 growth rates were high only in the first 96 h, indicating slightly better growth conditions in the beginning. This is when the provided urea is assumed to still be present in the medium, thus enabling unlimited exponential growth. These higher growth rates are also reflected in the DO levels at pH 4 and 6.7, where the DO decreases during the first 96 h, then increases to its initial level. After this first growth phase, the growth rates at pH 4 and 6.7 decreased rapidly and overall biomass formation was lower at pH 6.7, compared to pH 2.5, despite the availability of carbon source. These observations on the growth behavior are further discussed in section “Effect of C/N Ratio” and “CL Formation Kinetics in Shaking Flasks and 1 L Bioreactors,” in relation to the urea level in the fermentation medium.

When interpreting growth behavior, it is important to consider both backscatter and DO levels, because the backscatter signal, which is used as indicator for biomass concentration, can also be affected by CL crystals in the medium. However at pH 4 and 6.7, where no CL production occurred, the backscatter signal is assumed to result only from biomass in the media, while at pH 2.5, this signal may also be affected by CL concentration. This is shown in the higher CDW values at the end of fermentation at pH 4, compared to pH 2.5, although backscatter values were lower.

The obtained results show that the optimal pH value amongst the examined range for CL synthesis is at pH 2.5, which coincides with similar observations on other CL producing microorganisms. A pH range of 3–3.5 is reported to result in a threefold increase of product formation rate of CL by *U. maydis* compared to a pH range of 5–6 (Günther et al., 2010). This could partly be due to a decrease in product inhibition in this pH range. CL are known to precipitate in acidic pH ranges (Figure 1B), thus resulting in a decrease in CL concentration in the liquid medium (Haskins, 1950). Another cause for the observed results is likely to be due to stimulation of the secondary metabolism, caused by stress induced by the acidic pH range. However these results may vary, depending on the used fermentation media. The synthesis of CL observed by Geiser et al. (2014), on various *Ustilaginaceae* species for instance, occurred at pH 6.5. Therefore it can be assumed, that the pH dependency of CL formation is not only related to metabolic factors and stressors, but also to interactions between the produced CL and components present in the fermentation medium.

Considering the large impact of pH observed in this study, it would be interesting to further examine the effect of smaller variations in pH value (around pH 2.5) on CL productivity. Especially in regards to a potential CL fermentation in an industrial scale, more knowledge on pH sensitivity is crucial for process control. However, this observed highly acidic pH optimum is of great advantage regarding sterility aspects. At such low pH values, the maintenance of a sterile process is much easier compared to higher pH ranges, where contamination of the fermenter is more likely to happen.

### Effect of C/N Ratio

**FIGURE 5:**
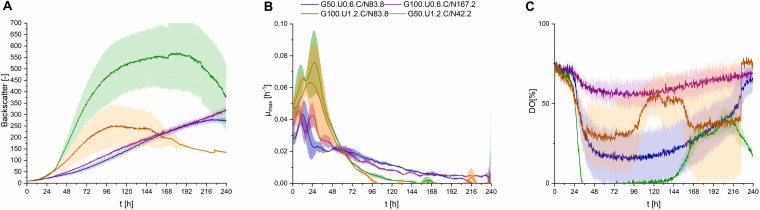
Observations on *S. scitamineum* during fermentation in the micro-bioreactor system, using PCM at an inoculation pH of 2.5, while glucose and urea concentrations were varied. Glucose concentration in the medium in g⋅L^–1^ is indicated as G, urea concentration in g⋅L^–1^ is indicated as U, while C/N ratio in mol⋅mol^–1^ is indicated as C/N. **(A)** Backscatter light signal, **(B)** specific growth rates, and **(C)** DO level. Error bars are deviated from biological duplicates or triplicates (*n* ≥ 2).

With the adjusted pH range of 2.5, three different C/N ratios were used in the fermentation medium, at different concentration levels. Both cultures with 1.2 g⋅L^–1^ urea [(I) 42.2 mol_C_⋅mol_N_^–1^ and (IIb) 83.8 mol_C_⋅mol_N_^–^1] showed higher maximum growth rates, while in cultures containing only 0.6 g⋅L^–1^ urea [(IIa) 83.8 mol_C_⋅mol_N_^–1^ and (III) 167.2 mol_C_⋅mol_N_^–^1] growth kinetics were slower in the first 24 h, indicating a direct relation of urea content in the fermentation medium to growth kinetics (Figures 5A,B). This was also reflected in the rapid decrease in DO level in the first 24–48 h (Figure 5C), compared to cultures with lower Urea concentrations, correlating to the shorter lag phase also observed in backscatter values.

This may be an indication, to two metabolic phases for *S. scitamineum*, the first occurring under nitrogen availability where growth is exponential and the second under nitrogen limited conditions, where a linear secondary growth phase occurs, as observed for *U. maydis* (Klement et al., 2012; Voll et al., 2012; Hartmann et al., 2018). Under nitrogen availability, the cells can metabolize glucose faster and thus show higher growth rates. This can be observed in the growth rates, where the highest growth rates remain over a longer time span for media containing 1.2 g⋅L^–1^ urea, compared to those with only 0.6 g⋅L^–1^. When nitrogen is depleted, cells shift to their secondary growth phase, where morphological changes and an increase in C/N ratio of the biomass probably occur, as previously observed for *U. maydis* (Klement et al., 2012; Voll et al., 2012). This increase in C/N ratio may be attributed to triglyceride accumulation in the cells, resulting in the morphological changes (Ageitos et al., 2011; Klement et al., 2012). A further cause for the increase in backscatter signal occurring in this secondary growth phase can be attributed to the utilization of internal nitrogen storage by the cells, while overall metabolism levels are lower, thus explaining the decreased oxygen uptake in the second growth phase, reflected in the increase in DO level. An increase in cell number, corresponding to the increase in CDW was observed for *U. maydis* even after nitrogen depletion, while the carbon source was still present in the media (Klement et al., 2012). In order to get more insight on these observations, nitrogen levels in the medium needed to be studied more intensively, which was performed in the bioreactor. Due to larger initial volumes, sampling was possible and the urea concentration in the medium was measured (section “CL Formation Kinetics in Shaking Flasks and 1 L Bioreactors”).

In regards to overall concentrations, higher biomass concentrations were obtained from media containing 1.2 g⋅L^–1^ urea, compared to the respective glucose concentrations with only 0.6 g⋅L^–1^ urea (Table 2). This may seem to contradict the lower end-backscatter values observed for media containing 1.2 g⋅L^–1^ urea, however, in that case the higher backscatter values observed for media containing only 0.6 g⋅L^–1^ urea are probably caused by an accumulated backscatter signal induced from both biomass and CL crystals.

At the lowest C/N ratio less than 0.5 g⋅L^–1^ CL was produced, while at the higher C/N ratio and the same amount of urea this amount increased to 2.1 ± 0.2 g⋅L^–1^. With the low level of 0.6 g⋅L^–1^ urea, maximum CL concentrations were obtained for both C/N ratios, with a c_CL_ of 5.1 ± 0.3 g⋅L^–1^ at 83.8 mol_C_⋅mol_N_^–1^ and 4.8 ± 1.2 g⋅L^–1^ at 167.2 mol_C_⋅mol_N_^–1^ (Table 2). As a result, low overall nitrogen concentrations together with a high C/N ratio seem to favor CL synthesis and were selected for further experiments in the bioreactor. At higher nitrogen concentrations, a larger amount of the available carbon source is used for biomass formation, before limitation occurs and the cells start producing CL. Therefore, for batch fermentation, high C/N ratios with low nitrogen concentrations are favored. However, if a fed batch process is designed, lower C/N ratios with feeding of carbon source may be considered. This would increase the available biocatalysators, thus enabling higher CL titers after feeding.

Furthermore, in media containing 100 g⋅L^–1^ glucose an increase in synthesized erythritol was observed, with concentrations up to 20 g⋅L^–1^. In media containing only 50 g⋅L^–1^ glucose, a maximum of 5 g⋅L^–1^ erythritol was measured. Erythritol is a known secondary metabolite produced by *S. scitamineum* and intermediate for the MEL synthesis (Peros et al., 1986). If erythritol production is not targeted, this effect of increased erythritol productivity at high initial glucose concentrations should therefore also be considered, when designing a fermentation process.

### Effect of Carbon Source

While nitrogen concentration revealed to be of importance for biomass formation and the induction of CL synthesis, the used carbon source may be of relevance for the produced CL titer. *S. scitamineum* is known to metabolize glucose, fructose and sucrose at similar rates (Peros et al., 1986), and *S. scitamineum* (NBRC 32730) produced MEL using all three carbohydrates (Morita et al., 2009a,b).

**FIGURE 6:**
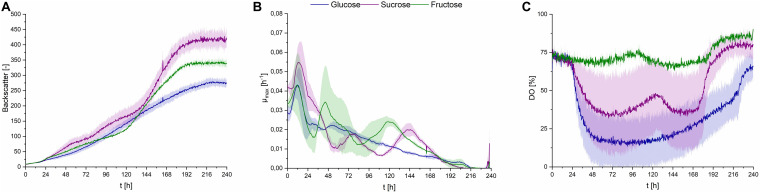
Observations on *S. scitamineum* during fermentation in the micro-bioreactor system, at an inoculation pH of 2.5, using PCM, 0.6 g⋅L^–1^ urea and 50 g⋅L^–1^ glucose, sucrose or fructose. **(A)** Backscatter light signal, **(B)** specific growth rates, and **(C)** DO level. Error bars are deviated from biological duplicates or triplicates (*n* ≥ 2).

Using all three substrates at a concentration of 50 g⋅L^–1^ in PCM, CL production was observed. Cells growing on sucrose showed higher growth rates, compared to glucose or fructose (Figures 6A,B). DO level barely decreased when fructose was the carbon source, compared to glucose and sucrose, where a decrease to up to less than 25% occurred (Figure 6C). This may be explained by the overall lower uptake rates of fructose, compared to glucose, when both sugars are present in the fermentation medium (Morita et al., 2015). This difference in oxygen uptake of *S. scitamineum* depending on the carbon source glucose or fructose, may further explain the unsteady DO levels when using sucrose as substrate, in which both sugars can be metabolized after hydrolysis of sucrose to both its constituents, glucose and fructose. However, in order to get a better understanding on the different growth behaviors depending on the carbon source, further studies analysing the carbohydrate concentrations in the medium and uptake rates during each fermentation phase need to be done. Nonetheless, all three carbon sources yielded similar amounts of biomass overall (Table 2), which corresponds to previous observations (Peros et al., 1986). The higher backscatter levels with sucrose may be explained by the comparably higher CL contents in these fermentations, which may interfere with scattered light measurement.

CL concentrations were slightly higher with sucrose, compared to glucose, resulting in a c_CL__,sucrose_ of 6.6 ± 0.2 g⋅L^–1^, c_CL__,glucose_ of 5.1 ± 0.3 g⋅L^–1^ and c_CL__,fructose_ of 6.1 ± 0.1 g⋅L^–1^, respectively (Table 2).

Similar results were found in this context by Günther et al. They observed an increase in CL concentrations with *U. maydis* when using sucrose as C source (Günther et al., 2010). Therefore, sucrose was selected as the most favorable carbon source for CL production and should be used in future experiments.

### Effect of Media Constituents

The screening media reported for *S. scitamineum* cultivation for CL synthesis by Geiser et al. (2014) consists of a mineral salt solution, trace elements and vitamins. For our cultivations we used the highly similar PCM medium. In order to determine the effect of its different constituents on CL synthesis we further examined a PCM medium without any trace elements (TE), containing only mineral salts and vitamins (PCM-TE) and a PCM medium without vitamins (Vit), containing only mineral salts and trace elements (PCM-Vit).

**FIGURE 7:**
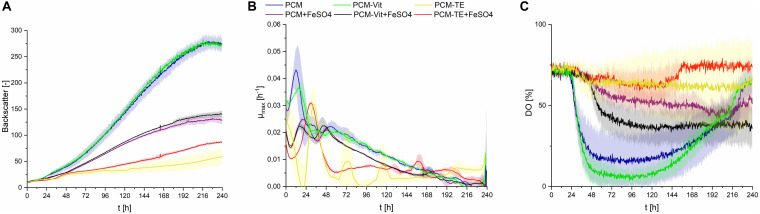
Observations on *S. scitamineum* during fermentation in the micro-bioreactor system, at an inoculation pH of 2.5, using 0.6 g⋅L^–1^ urea and 50 g⋅L^–1^ glucose, while varying the medium composition as indicated in the diagram. +FeSO_4_, addition of 0.01 g⋅L^–1^ FeSO_4_; –TE, PCM medium without trace elements; –Vit, PCM medium without vitamins. **(A)** Backscatter light signal, **(B)** specific growth rates, and **(C)** DO level. Error bars are deviated from biological triplicates (*n* = 3).

When provided only mineral salts and vitamins (PCM-TE), *S. scitamineum* showed very low growth rates and no CL production (Figures 7A,B and Table 2). The addition of iron resulted in an increase of biomass concentration c_CDW_ from 2.3 ± 0.5 g⋅L^–1^ to 3.5 ± 0.2 g⋅L^–1^, thus indicating a positive effect of iron addition on biomass formation. Iron was also observed to have positive effects on growth of *U. maydis* (Ardon et al., 1998). This was also reflected in slightly higher growth rates (Figure 7B). However, glucose was not completely metabolized in both media, after 240 h of fermentation, showing a general need of TE in the medium, for both growth and CL formation.

In PCM lacking vitamins (PCM-Vit) both biomass and CL concentrations were only slightly lower as when using the regular PCM (Table 2). Backscatter signal, DO level and growth rate curves were also comparable (Figure 7C). These results are in agreement with various studies, which showed that many yeasts, including *Ustilago maydis*, don’t need vitamins for their growth (Burkholder et al., 1944; Kurtzman and Fell, 2011). It can generally be assumed, that microorganisms showing a good growth behavior on media lacking vitamins, are able to synthesize the vitamins they need (Lindegren, 1945).

The addition of iron to these media however barely affected biomass formation, resulting in the same CDW concentrations after 240 h of fermentation, for both the regular PCM and PCM-Vit with or without iron. At the same time, a decrease in CL concentration was observed due to the addition of iron, from 3.8 ± 0.6 g⋅L^–1^ to 1.5 ± 0.1 g⋅L^–1^ for PCM-Vit, and from 5.1 ± 0.3 g⋅L^–1^to 1.4 ± 0.1 g⋅L^–1^ for PCM, respectively. This decrease in CL concentrations may partly explain the lower backscatter levels with media containing an excess of iron (Figure 7A), despite comparable CDW at the end of fermentation. However DO levels also increase when iron is added, indicating overall lower oxygen uptake. This may be explained by the probable decrease of activity of the monooxygenases Cyp 1 (CDU25005.1) and Cyp 2 (CDS01512.1) when less CL is produced, resulting in lower oxygen demand by the cells.

Overall, the results observed with or without an excess of iron in the media, show a direct impact of its concentration on both biomass growth and CL productivity. Iron concentration is known to affect glycolipid synthesis, as described for rhamnolipids (Guerra-Santos et al., 1984; Schmidberger et al., 2014; Shatila et al., 2020). Therefore, it is of high importance for an optimized CL production media, to determine the optimal iron concentration in the medium. We further showed that a minimal medium consisting only of mineral salts and trace elements, is sufficient for both growth of *S. scitamineum* and its CL synthesis. This is of high advantage when it comes to large scale production of CL. Vitamins are heat-labile and thus can only be sterilized via sterile filtration, as opposed to the used mineral salts and compounds supplying trace elements, that can be heat sterilized. This would save an additional sterilization step when designing a fermentation process.

### CL Formation Kinetics in Shaking Flasks and 1 L Bioreactors

**FIGURE 8:**
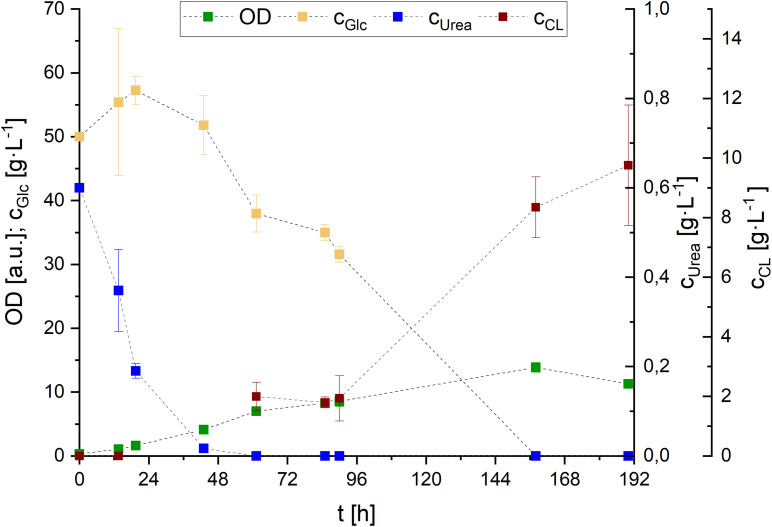
Glucose and urea consumption, as well as OD and CL concentrations during *S. scitamineum* fermentation in 1 L baffled shaking flasks in PCM with 0.01 g⋅L^–1^ FeSO_4_ at 30°C. Error bars are deviated from biological triplicates (*n* = 3).

In order to get a better understanding on CL formation kinetics and substrate uptake, we performed a cultivation with PCM including 0.01 g⋅L^–1^ FeSO_4_ and 50 g⋅L^–1^ glucose in shaking flasks (Figure 8). Based on the previously mentioned screening results, we fermented at a pH of 2.5 using 0.6 g⋅L^–1^ urea as nitrogen source. We used glucose as carbon source instead of sucrose, despite its better performance regarding CL productivity, in order to be able to further analyze growth behavior and CL synthesis in relation to nitrogen availability in the medium. Using sucrose would have added an additional factor that may affect the different growth behaviors/phases due to potentially different uptake rates of glucose and fructose, after sugar hydrolysis.

While growth rates remained in a relatively low range, with μ < 0.1 h^–1^ during the first 48 h and μ < 0.05 h^–1^ throughout the remaining growth phase, a maximum of 5.2 ± 0.1 g⋅L^–1^ biomass was formed after 162 h of cultivation. CL was detected in the medium after urea was completely consumed (∼48 h), and increased in concentration until glucose was entirely metabolized. Over the course of fermentation, CL concentration increased up to 8.3 ± 1.0 g⋅L^–1^ after 158 h.

**FIGURE 9:**
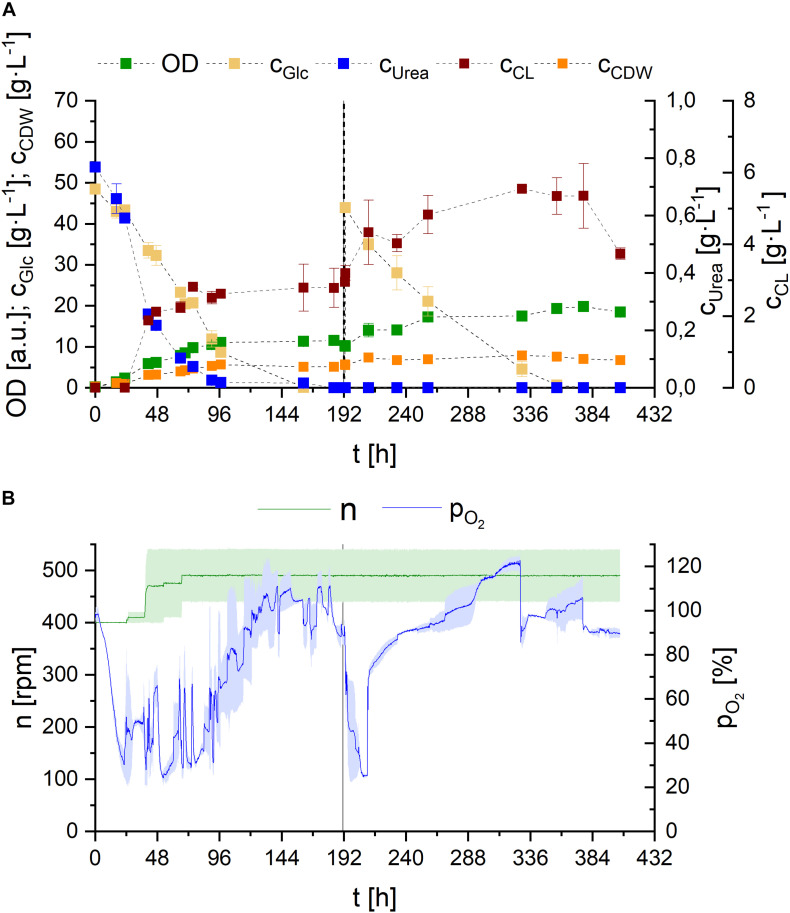
Observations on *S. scitamineum* fermentation in 1 L bioreactor in PCM with 0.01 g⋅L^–1^ FeSO_4_ at 30°C. **(A)** Glucose and urea consumption, as well as OD, CDW, and CL concentrations; and (B) DO level and stirrer speed. The dotted line indicates glucose feed. Error bars are deviated from biological duplicates (*n* = 2) and indicated as shaded area in **(B)**.

In order to be able to observe DO level to gain more information on the growth behavior of *S. scitamineum*, and to confirm the scalability of this shaking flask cultivation, we transferred the process to the bioreactor. There we added an additional feed of glucose (50 g⋅L^–1^) after it was completely consumed (190 h), since a higher glucose concentration proved to be beneficial for higher CL concentrations, as shown in the screening experiments in the micro-bioreactor. All other media constituents remained the same as in the shaking flasks. Fermentation in the bioreactor was associated with extreme foam formation, which explains the high noise observed in DO values that was caused due to pressure fluctuations during excessive foaming phases. The general growth kinetics were similar to the results obtained in shaking flasks (Figure 9A). OD and CDW increased during the first 161 h of fermentation, until which glucose was completely metabolized. Urea was consumed after 90 h of fermentation.

The concentration levels of glucose and urea correlate with the DO oxygen level in the reactor (Figure 9B), reinforcing the hypothesis of two distinct metabolic phases, previously mentioned in section “Effect of C/N Ratio.” Right after a short lag phase, glucose and urea are metabolized and OD, as well as CDW increase, while DO content decreases rapidly. To avoid oxygen limitation, stirrer speed needed to be increased from 400 rpm up to 500 rpm during the first 72 h of fermentation, corresponding to an increase in k_L_a value from 77 to 108 h^–1^ (values calculated internally). After 90 h of fermentation, when urea is consumed, a steady increase in DO level and thus decrease in oxygen consumption is observed, until the maximum is reached, when glucose is completely depleted. The glucose feed after 190 h is again reflected in an instant decrease in DO level to 20%. However here, the consequent increase to the maximum occurs faster.

These results, providing additional insight into substrate consumption, further emphasize the observations described in our micro-bioreactor cultivation with different urea concentrations, supporting the hypothesis that primary growth occurs when nitrogen is present in the medium. Cells then shift to secondary growth using internal nitrogen reserves and/or accumulate tryglycerides after nitrogen limitation. The production of glycolipids may be the result of overflow metabolism for the yeast, in order to regulate the intracellular energy level, when the depletion of a factor in the medium occurs, as hypothesized for sophorose lipid production by *Candida bombicola* and flocculosin secretion by *A. flocculosa* (Davila et al., 1997; Hammami et al., 2008).

However, opposed to our cultivations in shaking flasks, low amounts of CL were detected in the reactor, prior to complete urea depletion. Nevertheless higher CL concentrations were still occurring after nitrogen limitation. The effect of nitrogen on CL synthesis is controversially discussed in literature. While many describe nitrogen starvation as a direct trigger for CL synthesis, some observations state otherwise: In various basidiomycetous yeast strains, glycolipid formation is described to be induced by nitrogen starvation (Morita et al., 2013; Zavala-Moreno et al., 2014) and CL synthesis is generally suggested to be induced by nitrogen starvation in *U. maydis* (Frautz et al., 1986; Teichmann et al., 2007). Expression of Cyp1, the gene involved in hydroxylation of the fatty acid in CL, even revealed that it is induced by nitrogen starvation and detected with a delay of 24 h (Hewald et al., 2005). Gene sequence analysis of *S. scitamineum* identified a highly orthologous gene CDU25005.1. However gene expression analysis have to be performed to suggest similar regulation. Interestingly, there are also reports on CL production by *A. flocculosa* without nitrogen limiting conditions (Hammami et al., 2008).

Therefore, based on our observed results, we suggest more investigation on other nutrient limitations in the medium, like phosphorus, that may have appeared by that course of fermentation and are also involved in triggering CL synthesis.

Looking at the overall produced CL concentrations, we observed relatively low values in the medium, compared to shaking flask results. These can be explained by the produced foam and therefrom resulting deposits on the walls of the bioreactor. While a maximum CL concentration of 5.5 ± 0.1 g⋅L^–1^ was measured in the reactor, the remaining produced CL was deposited outside the liquid culture, transported by the foam. Balancing CL contained in these deposits after termination of the fermentation resulted in an overall produced CL amount of 10.6 ± 1.2 g, corresponding to a concentration of 17.6 g⋅L^–1^ in the final culture broth, at purities ranging from 85 to 93%, depending on the purified fraction. Overall higher purities were observed in the fractions obtained from the foam deposits, compared to CL directly extracted from the culture broth. Based on the screening results in sections “Effect of Carbon Source” and “Effect of Media Constituents,” these concentrations could further increase, if sucrose is used as carbon source and is therefore recommended for future CL fermentations with *S. scitamineum*.

### MALDI-TOF MS Analysis of the Produced Cellobiose Lipid Structures

**FIGURE 10:**
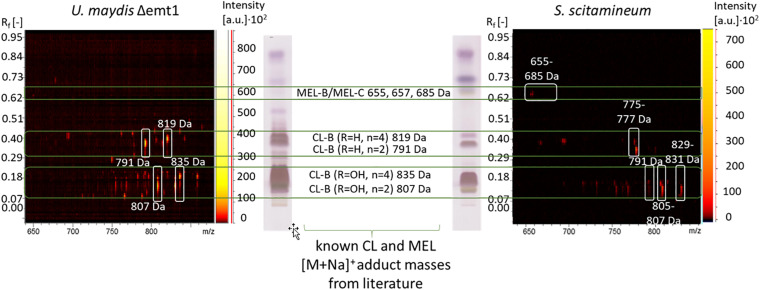
Procedure of CL structure determination via HPTLC coupled MALDI-TOF-MS analysis. The polarity pattern of an HPTLC lane of a CL extract obtained from a *S. scitamineum* fermentation is compared with the polarity pattern of a CL extract from *U. maydis* (DSM 17146) with known CL structures. MEL structures have higher retardation factors R_f_ due to their higher hydrophobicity. In combination with the polarity pattern, known CL and MEL masses are compared with the masses of [M + Na]^+^ adducts from the 2-dimensional m/z spectrum of the scanned lane, showing all obtained masses. Only spots with an intensity above the threshold of 500 a.u. are considered. The identified masses of CL and MEL variants produced by *S. scitamineum* are highlighted with white squares in the 2-dimentsional m/z spectrum. All detected masses of both TLC lanes are presented in Supplementary Tables 1, 2.

To determine the CL structures produced by *S. scitamineum*, a sample of an ethanol extract was separated due to polarity on an HPTLC plate and compared to an HPTLC lane pattern of *U. maydis* (DSM 17146) CL. CL-B structures with one or two hydroxyl groups in the fatty acid chain have lower retardation factors R_f_, compared to CL-B structures without any additional hydroxyl groups (Figure 10). [M + Na]^+^ adduct masses of 807 Da were identified at an R_f_ range of 0.08–0.18, corresponding to the CL-B variant with the acylated cellobiose moiety linked to a 2,15,16-trihydroxy-hexadecanoic acid via its ω-hydroxyl group, with *n* = 2 and R_1_ = OH. This detected CL-B variant corresponds to one of the structures also identified by Deinzer (2017) with another *S. scitamineum* strain and already known for *U. maydis* CL (Spoeckner et al., 1999; Teichmann et al., 2011b). Other variants with Δ2 Da each, corresponding to 805 Da were also detected, indicating an unsaturated fatty acid chain. This could explain the slight difference in R_f_ values between *S. scitamineum* CL and *U. maydis* CL, typically observed when the characteristic HPTLC lanes of CL extracts from both microorganisms are compared. m/z differences of Δ16 Da were attributed to additional hydroxyl groups. Masses of 791 Da were also detected, however, the absence of the hydroxyl group at R_1_ should have resulted in higher R_f_ values here, as can be seen in the *U. maydis* TLC lane (0.21–0.4). With R_f_ values in the same range as the masses with 807 Da, the absence of the hydroxyl group in this case has to be at another position than R_1_. Differences of Δ28 Da correspond to the length of the acetyl chain *n* = 2 or 4 and result in the values of the less polar variants with 835 and 819 Da produced by *U. maydis*. These masses were not detected in the TLC lane of *S. scitamineum*, however a polar variant with 829–831 Da was identified in high quantities, at the same R_f_ range of the 835 Da variant from *U. maydis*. The difference of 4 Da here may be explained by the presence of a di-unsaturated fatty acid, which again would explain slight differences in R_f_ values between *S. scitamineum* CL and *U. maydis* CL TLC lanes. A less polar variant with 775–777 Da was detected in the Rf range of the CL-B variant with R_1_ = H and *n* = 2 (791 Da) of *U. maydis*. The mass difference of Δ14 Da of the 777 Da variant may indicate the presence of a C15 fatty acid chain in this more hydrophobic CL variant of *S. scitamineum*, while the difference of Δ16 Da of the 775 Da variant would be attributed to a missing hydroxyl group. This may be an indication of a variant without a hydroxyl group at the C6 fatty acid, as was desccribed for Δuhd1 mutants of *U. maydis*, or a missing hydroxyl group at the C16 fatty acid, as was described for Δcyp2 mutants of *U. maydis* (Teichmann, 2009). Both hypothetical variants would result in a slight change in R_f_ values of the detected TLC lanes of CL samples from *S. scitamineum* compared to the CL samples from *U. maydis*, as is typically observed here. However for a more detailed structure determination of these detected CL masses, further analyses like MSMS need to be done.

Several other masses in the mass spectrum range between known CL and MEL structures (685 – 775 Da) were also detected, however not yet identified (see detailed in Supplementary Table S1). We further identified less polar structures with m/z of 655 Da, 657 and 685 Da that correspond to known MEL-B/MEL-C structures (Deinzer, 2017; Beck et al., 2019).

## Conclusion

We here report the first systematic description of factors affecting CL synthesis by *S. scitamineum* leading to high CL concentrations, up to 17.6 g⋅L^–1^ in a 1 L bioreactor. A pH of 2.5, a C/N ratio of 83.8 mol_C_⋅mol_N_^–1^ using 0.6 g⋅L^–1^ urea as nitrogen source and sucrose as carbon source proved to be optimal for CL synthesis, while vitamins were not essential for glycolipid production by *S. scitamineum.* Nitrogen and iron concentrations, however, have a major effect on both biomass growth and CL formation and should be considered for further media optimization.

Via a TLC coupled MALDI-TOF MS method we identified the produced CL structures as a mixture of different CL-B variants. which correspond to some of the known CL structures that are produced by *U. maydis*. This is explained by the highly similar CL synthesis gene cluster we identified by BLAST analysis in the published genome sequence data of *S. scitamineum* SscI8. Furthermore, the sequences of the identified CL-biosynthesis cluster can be used in future studies to complement the presented work by gene expression data to achieve further insights into the regulation of the CL biosynthesis in *S. scitamineum*.

## Data Availability Statement

Publicly available datasets were analyzed in this study. This data can be found here: ASM90000236v1.

## Author Contributions

AO contributed conception and design of the study, analysis and interpretation of the data, and wrote the first draft of the manuscript. NW contributed with gene sequence analysis and interpretation. ZS assisted with the experimental work. SZ acquired grants and supervised AO in her work, and contributed to scientific arrangement and manuscript revision. All authors read and approved the submitted version.

## Conflict of Interest

The authors declare that the research was conducted in the absence of any commercial or financial relationships that could be construed as a potential conflict of interest.
