# Supplementary material for: Factors Affecting the Synthesis of Cellobiose Lipids by Sporisorium scitamineum
Source: Front Bioeng Biotechnol. 2020 Nov 4;8:555647. doi: 10.3389/fbioe.2020.555647 (PMC7673458; doi:10.3389/fbioe.2020.555647)
Supplement: Supplementary file 1 [file Data_Sheet_1.PDF]

Supplementary Material

1 Supplementary Figures and Tables

Supplementary Table 1. Identified masses from the 2-dimensional m/z spectrum of an HPTLC lane of *S. scitamineum* CL extract, detected via MALDI-TOF-MS. Only masses that correspond to the polarity pattern of the HPTLC lane (R_f values in Fig. 10) with an intensity above 500 a.u. (threshold) and in the mass range of known CL or MEL structures are presented. Identified CL adduct masses are indicated in blue and MEL adduct masses in green, while corresponding masses with a difference of 1 Da and an intensity above the threshold, attributed to the natural isotopic distribution of the atoms, are indicated in light blue and light green.

Running distance [mm]	m/z of $[M+Na]^+$ [Da]	Intensity [a.u.]	R_f [-]
160	769.97	686	0.08
	807.45	873	
	823.43	2209	
	824.39	946	
	824.39	946	
	845.29	1173	
166	807.27	819	0.09
	823.25	844	
172	807.29	862	0.10
178	805.24	747	0.11
	807.26	2232	
	808.25	928	
	827.22	1195	
	829.23	1599	
184	754.01	602	0.12
	754.05	635	
	807.27	4069	
	808.27	1507	

	829.26	1945	
	830.27	884	
190	753.95	1180	0.13
	754.91	588	
	755.07	580	
	767.59	943	
	807.21	7303	
	808.22	2945	
	809.25	921	
	829.19	3788	
	830.21	1316	
196	753.94	1386	0.14
	754.91	702	
	767.60	1468	
	807.22	9911	
	808.26	4204	
	809.25	1351	
	829.22	5044	
	830.24	2002	
	831.26	573	
202	753.94	1613	0.15
	754.95	696	
	761.27	638	
	767.60	1350	
	768.59	585	
	807.21	9813	
	808.22	4250	
	809.23	1267	
	829.19	5169	
	830.19	2212	
	831.19	574	

208	731.24	930	0.16
	731.38	840	
	751.97	756	
	805.24	4614	
	806.22	1547	
	807.24	3378	
	808.26	1060	
	829.24	1594	
214	693.18	642	0.18
	715.26	781	
	731.33	1389	
	751.91	857	
	752.02	952	
	765.69	718	
	765.83	733	
	791.28	1181	
	805.25	4929	
	806.24	2060	
	807.31	714	
	813.25	787	
220	791.25	1627	0.19
	792.25	709	
	805.26	1178	
	813.23	858	
292	777.34	669	0.32
298	777.29	2622	0.33
	778.29	1133	
304	777.24	7184	0.34
	778.25	2886	

	779.27	768	
	799.21	708	
310	777.21	13742	0.35
	778.23	5686	
	779.22	1687	
	799.23	1305	
316	777.23	11884	0.36
	778.24	5013	
	779.26	1274	
	799.20	1233	
	800.19	496	
322	777.25	856	0.37
	777.41	709	
328	666.49	1259	0.38
	667.53	597	
	692.50	660	
	694.53	577	
	775.20	1160	
	776.36	446	
334	666.50	1406	0.40
	667.49	718	
	692.50	3482	
	693.49	1730	
	694.55	2874	
	695.60	1278	
	775.23	2975	
	776.26	1059	
	777.27	679	
	777.38	607	

340	692.53	3195	0.41
	693.53	1382	
	694.53	2632	
	695.56	1061	
	761.29	855	
	775.27	2056	
	776.27	702	
466	608.29	784	0.64
	629.34	780	
	655.33	1135	
	655.46	938	
	657.36	1085	
472	655.26	1257	0.65
	656.30	455	
	657.26	2111	
	658.34	747	
478	608.26	536	0.66
	655.37	772	
	657.34	1835	
	658.36	728	
	685.43	676	

Supplementary Table 2. Identified masses from the 2-dimensional m/z spectrum of an HPTLC lane of *U. maydis* Δemt1 CL extract, detected via MALDI-TOF-MS. Only masses that correspond to the polarity pattern of the HPTLC lane (R_f values in Fig. 10) with an intensity above 500 a.u. (threshold) and in the mass range of known CL structures from *U. maydis* are presented. Identified CL adduct masses are indicated in blue, while corresponding masses with a difference of 1 Da and an intensity above the threshold, attributed to the natural isotopic distribution of the atoms, are indicated in light blue.

Running distance [mm]	m/z of [M+Na]+ [Da]	Intensity [a.u.]	Rf [-]
160	787,38	826	0,08
172	765,40	1945	0,10
	766,37	604	
	787,37	1290	
	793,46	706	
178	793,50	1047	0,11
	807,47	903	
	815,45	699	
184	807,40	2588	0,12
	808,41	1020	
	829,43	1356	
	830,39	533	
190	807,44	3676	0,13
	808,43	1648	
	829,44	2048	
	830,40	689	
196	807,44	3676	0,14
	808,43	1648	
	829,44	2048	
	830,40	689	
202	807,44	3676	0,15
	808,43	1648	
	829,44	2048	
	830,40	689	
208	807,44	3676	0,16

	808,43	1648	
	829,44	2048	
	830,40	689	
214	807,42	7756	0,18
	808,44	3172	
	809,43	1137	
	829,41	3996	
	830,41	1742	
	835,45	4074	
	835,89	2379	
	836,47	1624	
	836,92	1073	
	857,44	1897	
220	782,11	672	0,19
	795,81	597	
	807,38	2779	
	808,40	890	
	829,36	1194	
	835,41	4305	
	836,41	1813	
	837,40	402	
	857,41	1884	
	858,36	745	
226	835,42	7963	0,20
	836,43	3759	
	857,42	3853	
	858,41	1832	
232	782,14	1404	0,21
	783,12	733	
	789,56	768	
	791,53	2756	
	792,53	1155	
	795,83	1140	

	813,52	634	
	819,57	877	
	819,86	613	
	835,53	6498	
	836,56	3300	
	837,61	991	
	851,54	638	
	857,51	2174	
	858,55	788	
	858,55	788	
238	791,43	963	0,22
	792,45	424	
	813,41	563	
	819,49	1052	
	841,46	676	
292	791,47	659	0,32
298	791,57	504	0,33
304	791,57	504	0,34
310	791,44	3654	0,35
	792,44	1570	
316	791,40	8366	0,36
	792,41	3074	
	793,44	993	
	813,38	652	
322	791,37	10747	0,37
	792,39	4420	
	793,41	1371	
	813,37	1175	
328	791,41	13433	0,38
	792,43	5692	
	793,41	1801	
	805,46	579	

	813,41	1504	
	819,48	2527	
	820,45	1078	
334	791,46	3216	0,40
	792,46	1161	
	805,46	1142	
	819,44	10151	
	820,45	4593	
	821,46	1399	
	841,43	1105	
340	805,42	781	0,41
	819,41	17580	
	820,42	7804	
	821,44	2463	
	841,38	1501	
346	775,59	1177	0,42
	819,53	7413	
	820,56	3742	
	821,55	1272	
352	775,39	1614	0,43
	776,39	681	
358	775,51	521	0,44
	835,44	576	
370	819,40	570	0,46
376	819,43	929	0,47
382	819,42	706	0,48
388	819,42	706	0,49

1.1 Supplementary Figures

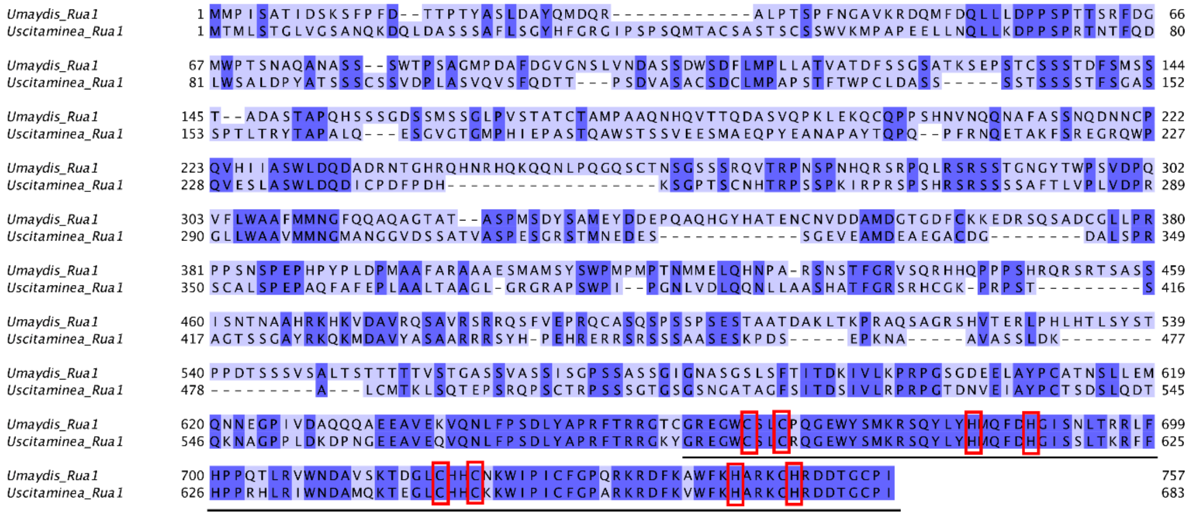

Supplementary Figure 1. Protein sequence alignment of *U. maydis* (NCBI: txid237631 Rua1 and its homologue in *S. scitamineum* (NCBI: txid1447027). Dark blue indicated high conserved amino acids in all sequences; blue indicates similar amino acids and light blue less similar amino acids. The Cys2His2-motif is marked with red boxes. The black underlined C-terminal region shows an amino acid sequence identity of 86.7 % and similarity of 92.9 %. The alignment was generated with the Jalview software. Identity and similarity of the amino acid sequence was determined using the EMBOSS Needle software (Madeira *et al.* 2019).
